# NASH-CHECK patient-reported outcome instrument: evaluation of content and face validity for patients with metabolic dysfunction–associated steatohepatitis and compensated cirrhosis

**DOI:** 10.1186/s41687-025-00881-6

**Published:** 2025-07-01

**Authors:** Lynda C. Doward, Matthew Breckons, Maria-Magdalena Balp, James Twiss, Luke Vale, Lorraine McSweeney, Clifford A. Brass, Quentin M. Anstee, Arun J. Sanyal

**Affiliations:** 1 RTI Health Solutions, Manchester, UK; 2 Population Health Sciences Institute, Faculty of Medical Sciences, Newcastle University, Newcastle Upon Tyne, UK; 3 Novartis Pharma AG, Basel, Switzerland; 4 Novartis Pharmaceutical Corporation, East Hanover, NJ USA; 5 Translational and Clinical Research Institute, Faculty of Medical Sciences, Newcastle University, Newcastle Upon Tyne, UK; 6 Newcastle NIHR Biomedical Research Center, Newcastle Upon Tyne Hospitals NHS Trust, Newcastle Upon Tyne, UK; 7 Stravitz-Sanyal Institute for Liver Disease and Metabolic Health, Virginia Commonwealth University School of Medicine, Richmond, VA USA

**Keywords:** Metabolic dysfunction–associated steatohepatitis, Burden, Health-related quality of life, Patient-reported outcomes, Psychosocial impact, NAFLD, Non-alcoholic steatohepatitis, NASH

## Abstract

**Background:**

NASH-CHECK is a patient-reported outcome measure (PROM) that assesses symptoms and broader health-related quality of life (HRQOL) impacts of metabolic dysfunction–associated steatohepatitis (MASH), previously called non-alcoholic steatohepatitis (NASH). The measure was initially developed and validated for patients with non-cirrhotic MASH. This study describes an evaluation of the suitability of NASH-CHECK for patients with compensated cirrhotic MASH.

**Methodology:**

Concept elicitation (CE) interviews were conducted with patients with clinically confirmed compensated cirrhotic MASH in the United States (US) and United Kingdom (UK) to determine the symptom burden and broader HRQOL impact of MASH. Symptoms and broader HRQOL impacts identified during analysis of the CE data were compared with the key concepts included in NASH-CHECK; any symptoms reported in the CE interviews but not reflected in NASH-CHECK were reviewed for relevance by clinical experts. The content validity of NASH-CHECK was evaluated further via cognitive debriefing (CD) interviews conducted with patients with compensated cirrhotic MASH in the US and UK.

**Results:**

CE interviews were conducted with 33 patients with compensated cirrhotic MASH (US = 9, UK = 24; 60.6% female; mean age, 64.3 years). Key symptoms described were similar to those reported by patients with non-cirrhotic MASH identified during the development of NASH-CHECK; these included abdominal pain, abdominal bloating, itch, fatigue, sleeping difficulties, and cognitive symptoms. Other key HRQOL impacts included activity limitations and emotional, social, relationship, and work impacts. All key symptom and broader HRQOL impacts reported by patients with compensated cirrhotic MASH are currently included in NASH-CHECK, and no additional symptoms or HRQOL impacts reported during the CE interviews were deemed relevant for inclusion. CD interviews were conducted with 17 patients with compensated cirrhotic MASH (US = 8, UK = 9; 47.1% female; mean age, 62.8 years). Patient feedback on NASH-CHECK content confirmed that the concepts captured by the items were considered important, relevant, and comprehensive for addressing the impact of compensated cirrhotic MASH.

**Conclusions:**

The results support the content validity of NASH-CHECK for patients with compensated cirrhotic MASH, demonstrating that NASH-CHECK is a suitable PROM for use in clinical trials, studies, and practice for this patient population.

**Supplementary Information:**

The online version contains supplementary material available at 10.1186/s41687-025-00881-6.

## Abstract (plain language summary)

The condition widely known as non-alcoholic steatohepatitis (NASH) has recently been renamed to “metabolic dysfunction–associated steatohepatitis” (MASH). This article uses the name “MASH” throughout. MASH causes progressive inflammation of the liver that can lead to damage, severe scarring of the liver (“cirrhosis”), and ultimately, liver cancer. Researchers are interested in understanding the experiences of people living with MASH, including the symptoms and daily life impacts of MASH. NASH-CHECK is a questionnaire that was first designed for people with the early stages of MASH, without severe scarring of the liver, to describe their symptoms and the impacts that MASH has on their quality of life. The purpose of this study was to understand whether NASH-CHECK is appropriate for people with MASH that has progressed to the first stage of severe liver scarring (“compensated cirrhotic MASH”). After interviewing 33 people in the United States and the United Kingdom to understand their experiences with MASH without severe liver scarring and after consulting with doctors who treat people with MASH, the researchers found that NASH-CHECK was suitable for people with MASH that has progressed to the first stage of severe liver scarring. NASH-CHECK then was tested with 17 people with compensated cirrhosis due to MASH, who confirmed that the questionnaire allowed them to describe the symptoms and impacts they experienced from MASH. People with MASH that has progressed to the first stage of severe liver scarring can use NASH-CHECK to evaluate the symptoms and impacts of MASH, which will help doctors and researchers to understand how people with this disease feel and function.

**Supplementary Information:**

The online version contains supplementary material available at 10.1186/s41687-025-00881-6.

## Background

Metabolic dysfunction–associated steatotic liver disease (MASLD) is characterised by a build-up of excess fat in the liver, which is comparable to the build-up seen in alcoholic liver disease, but in individuals who do not excessively consume alcohol [1–3]. Metabolic dysfunction–associated steatohepatitis (MASH) (formerly known as non-alcoholic steatohepatitis [NASH]) is the progressive and most severe form of MASLD and develops in approximately 25%–30% of patients with MASLD [4]. Progression to MASH is characterised by inflammation of the liver, with evidence of liver cell injury and different degrees of scarring or fibrosis [5]. Five fibrosis stages are used to categorise the severity and progression of MASH: Stage 0 (F0) indicates no fibrosis; Stage 1 (F1), enlargement of the portal areas by fibrosis; Stage 2 (F2), fibrosis extending from the portal areas, with rare bridges between portal areas; Stage 3 (F3), many bridges of fibrosis that link portal and central areas of the liver; and Stage 4 (F4), cirrhosis [6]. Cirrhosis may be classified as compensated or decompensated. In *compensated cirrhosis*, liver damage is present but the liver continues in its ability to perform its usual functions. Clinically significant elevated blood pressure in the liver’s blood vessels (portal hypertension) may be present, but the typical symptoms associated with decompensated cirrhosis, including overt buildup of fluid in the abdomen (ascites); confusion, disorientation, and changes in brain function resulting from a buildup of toxins in the brain (hepatic encephalopathy); and bleeding from dilated veins in the gastrointestinal tract (variceal hemorrhage), have not developed [7, 8]. *Decompensated cirrhosis* describes a stage of progression in which patients experience increased liver dysfunction and have a significantly increased risk of mortality [9, 10]. The median survival of patients with compensated cirrhosis is approximately nine to 12 years, whereas the median survival among patients with decompensated cirrhosis drops considerably to approximately two years [8, 9].

Although MASH historically was considered asymptomatic, even in advanced stages, evidence has shown that patients may experience burdensome, non-specific symptoms such as abdominal pain or discomfort, feelings of fullness, fatigue, cognitive impairments, and physical weakness [11–15]. Symptoms related to MASH can ultimately result in an impaired health-related quality of life (HRQOL)—that is, a patient’s experience of the impact of a disease or treatment on their ability to conduct their day-to-day activities, as well as the impact on their physical, social, and emotional functioning [11, 15]. Further, HRQOL has been reported to be lower among individuals with compensated cirrhotic MASH than among the general population and patients with non-cirrhotic MASH, particularly with respect to physical functioning and emotional and physical well-being [16]. The chronic-liver-disease–specific patient-reported outcome measures (PROMs) commonly used to evaluate HRQOL in patients with compensated cirrhotic MASH were not developed in accordance with industry or regulatory standards for PROMs and/or are not validated for use in a population with compensated cirrhotic MASH [16]. The limitations of the PROMs that have been used in studies evaluating the HRQOL impact of compensated cirrhotic MASH present a barrier to understanding the patient-perceived burden of this condition.

**Fig. 1:**
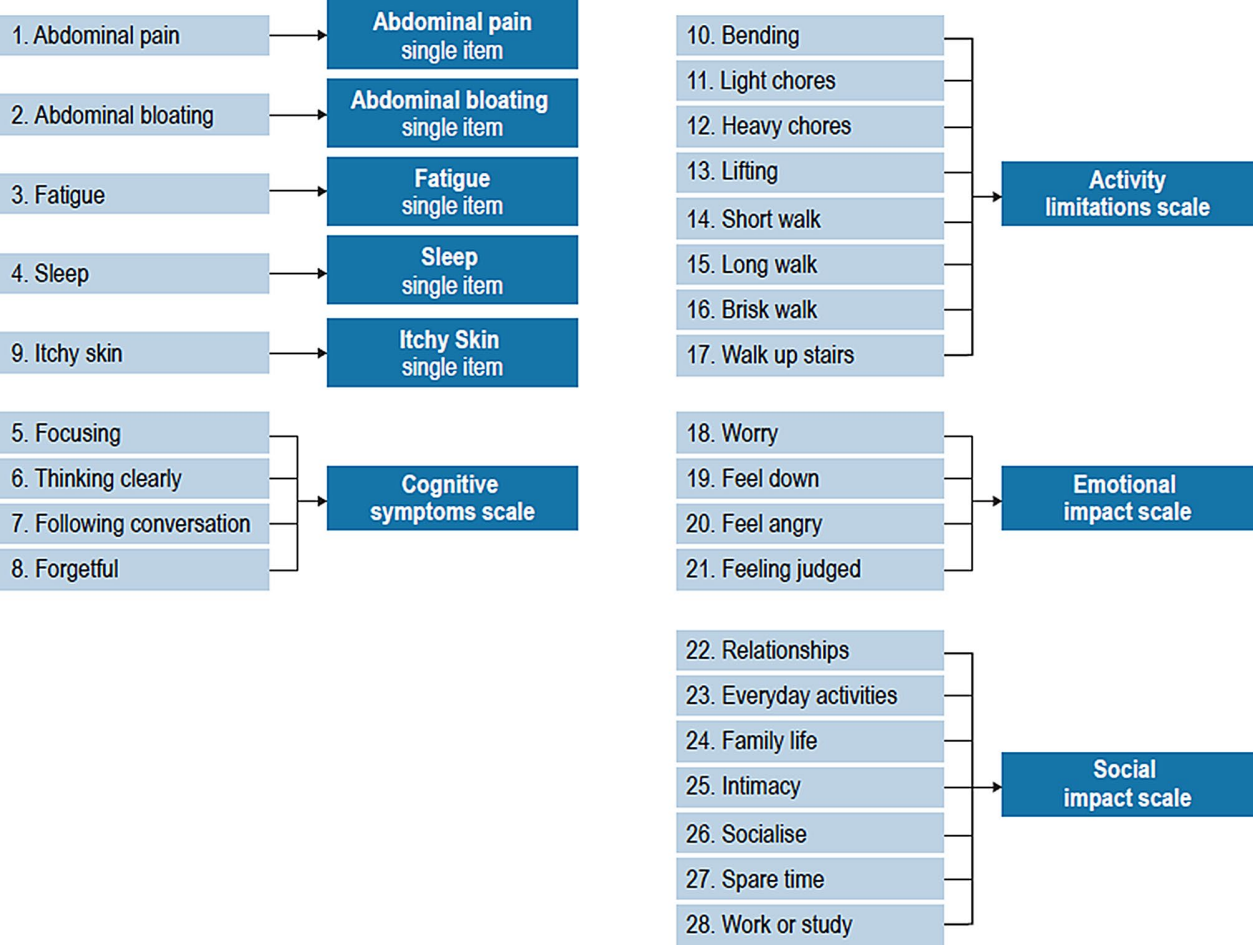
NASH-CHECK structure

NASH-CHECK is a novel PROM that was initially developed to assess symptoms and broader HRQOL impacts for patients with non-cirrhotic MASH (F1-F3) (Fig. 1). NASH-CHECK has been adopted as the patient-reported outcome (PRO) measure to inform the patient experience of individuals enrolled in the European MASLD Registry [17], which supports the work of the European Union IMI2–funded “Liver Investigation: Testing Marker Utility in Steatohepatitis” (LITMUS) consortium. LITMUS aimed to establish a defined set of biomarkers that, singly or in combination, enable detection and monitoring of liver disease progression (or regression) from MASLD, through MASH, to fibrosis and cirrhosis [18].

The development of NASH-CHECK comprised two phases. A qualitative development phase involving 23 concept elicitation (CE) interviews and 20 cognitive debriefing (CD) interviews with patients with F1-F3 MASH established the content validity of the measure for a non-cirrhotic patient population, demonstrating the measure to be relevant, comprehensive, and acceptable to patients [11]. A quantitative validation phase further supported the reliability, construct validity, and responsiveness of the measure (i.e., its ability to detect change) in a non-cirrhotic MASH patient population [19]. The methodology employed was consistent with best practices and with regulatory guidance on the development and validation of PROMs [20–22]. The final version of NASH-CHECK comprises 28 items assessing symptoms (9 items) and HRQOL impacts (19 items) [19].

As argued above, to improve understanding of the impact of compensated cirrhotic MASH from the patient perspective, fit-for-purpose PROMs that have been validated for this population are needed. The aim of this study was to rigorously evaluate the suitability of NASH-CHECK for patients with compensated cirrhotic MASH (F4). As advancement to compensated cirrhosis could lead to a different symptom experience compared with non-cirrhotic MASH, concept elicitation interviews were conducted with patients with compensated-cirrhotic NASH to assess the symptoms and broader HRQOL impacts experienced by this population. Following this, a clinical expert review stage was completed to evaluate the key symptoms identified from the concept elicitation interviews to evaluate if changes to the NASH-CHECK content was required. Finally, cognitive debriefing interviews were conducted with patients with compensated cirrhotic MASH to evaluate the content validity of NASH-CHECK for this patient population.

## Methods

The study consisted of three phases, described in detail in the sections that follow: CE interviews, clinical and research team review, and CD interviews. The study was conducted in accordance with the Declaration of Helsinki, and all participants provided informed consent before taking part in the study. The Western Institutional Review Board approved the research conducted with patients in the US. The London-Stanmore Research Ethics Committee provided National Health Service (NHS) ethics approval for the research conducted with UK participants, and the Health Research Authority granted permission to conduct the study (reference: 18/LO/1594).

### Study population

Qualitative interviews were conducted with adults with a diagnosis of compensated cirrhotic MASH (F4) in the US and UK. In the US, participants were recruited from Virginia Commonwealth University (VCU) clinics through the screening of clinic attendees’ medical records by a member of the VCU team. In the UK, participants were recruited from gastroenterology and hepatology clinics at Newcastle upon Tyne Hospitals NHS Foundation Trust. Patients were identified via purposive recruitment through either (1) the screening of clinic attendees’ medical records by a member of the medical care team or (2) their enrolment in the European MASLD Registry [17]. Identified people were approached to determine their interest in participation in the study by a mixture of methods that included email and face-to-face methods.

To be eligible for the study, patients had to be aged 18 to 75 years; have MASH cirrhosis (either histologically confirmed, or with clinical suspicion of progression to MASH cirrhosis based on clinical assessment and/or use of non-invasive tests/imaging); have alanine aminotransferase (ALT) and aspartate aminotransferase (AST) levels ≤ 10 times the upper limit of the normal range; and be able to read and understand English in order to provide informed consent and complete a questionnaire. Patients were ineligible if they had pre-cirrhotic MASH or other liver disease; had evidence of hepatic decompensation or hepatocellular carcinoma; had lost > 10% body weight in the previous year; had excessive alcohol consumption (on average, > 20 g per day in females and > 30 g per day in males) for a period longer than three consecutive months in the previous year; had active substance misuse; had severe mental illness that would affect their ability to provide informed consent or complete a questionnaire; had extreme obesity (body mass index [BMI] > 45); or had a Model for End-stage Liver Disease (MELD) score > 15.

### Concept elicitation interviews

In-depth CE interviews were conducted with patients in the US and UK to determine the patient-perceived impact of MASH and determine whether any MASH-related concepts relevant to patients with compensated cirrhosis (F4) were missing from NASH-CHECK. Interviews were conducted face to face, each by two experienced qualitative researchers (a lead interviewer and a notetaker) and were audio-recorded and transcribed. All interviewers had substantial experience in conducting qualitative research and were qualified to PhD level. Interviews were conducted for each participant at a single time point. Interviews in the US were conducted by two female researchers: TMB, a senior director, and CS, a director of Patient-Centered Outcomes (see Acknowledgements). Interviews in the UK were conducted by one male (MB) and one female (LMS) research associate from Newcastle University. No relationships were established between the researcher and the participant prior to the interview. Interviewees were informed as part of the consenting process that the interviews would be conducted by researchers from RTI Health Solutions (for US-based interviews) or Newcastle University (for UK-based interviews) who are not involved in their medical care and support. US-based interviews were conducted in a dedicated meeting space located in a hotel facility close to the VCU clinical site. A majority of the UK-based interviews were conducted at the participant’s home, with others conducted in meeting room spaces at Newcastle University, depending upon the preference of the interviewee.

Each interview lasted approximately one hour and followed a semi-structured CE interview guide that was based on existing understanding of the possible symptoms and broader HRQOL impacts of MASH but was flexible in its exploration of additional symptoms and impacts (see Supplemental Appendix A). Participants initially completed a demographic and disease background questionnaire and then, during the interviews, were asked about their MASH history, their experiences with MASH symptoms, and the impacts they experienced from MASH, including impacts on their day-to-day life, their emotional well-being, and their relationships.

### Clinical expert review of concept elicitation findings

Following completion of the CE interviews, the symptoms reported by the sample of patients with compensated cirrhotic MASH were compared with concepts that had been reported by the sample of patients with non-cirrhotic MASH (F1-F3) interviewed during the initial development of NASH-CHECK [11] and with the content of the final version of NASH-CHECK. Any new symptoms emerging from the interviews—that is, not previously reported during the earlier non-cirrhotic MASH (F1-F3) interview analysis and not included in NASH-CHECK—were reviewed and discussed by a group of international clinical experts at a face-to-face workshop during the general assembly meeting of the LITMUS consortium held on 17 October 2019. The clinical experts included five hepatologists working in clinical practice across Europe, responsible for treating patients with MASH, and a hematologist and a gastroenterologist based in the pharmaceutical sector. All were active contributors to the LITMUS program. Additional workshop participants included pharmaceutical representatives (health economics outcomes researchers and COA specialists) engaged in MASH-related programs. The meeting was facilitated by a COA experts (LD), a pharmaceutical representative (MMB), and an academic representative (MB). The symptoms were also reviewed by the lead clinical authors, QMA and AJS. The purpose of the review was to find consensus on the potential relevance of any new symptoms for inclusion in a PROM intended for use in compensated cirrhotic MASH.

### Cognitive debriefing interviews

Cognitive debriefing interviews were conducted with patients with compensated cirrhotic MASH in the US and UK to evaluate the content validity of NASH-CHECK for this patient population. The cognitive debriefing interviews were designed to assess the clarity, comprehensibility, understandability, and relevance of NASH-CHECK instructions and items, response options, and recall period specifically for a compensated cirrhotic MASH patient population. The interviews were conducted by the same experienced qualitative researchers who conducted the CE interviews, following a CD guide (see Supplemental Appendix B). Each interview was conducted by two researchers (a lead interviewer and a notetaker) and were audio-recorded and transcribed. Each interview lasted 45 min to one hour and followed a semi-structured CD interview guide. Interviews in each country were intended to be conducted face to face, with written consent obtained by the lead interviewer. However, due to the COVID-19 outbreak, it was necessary to conduct three interviews virtually via telephone using a verbal consent procedure. The face-to-face interviews were conducted at the same locations in the US and the UK as for the CE interviews. Participants initially completed the same demographic and disease background questionnaire used in the CE interviews. Then, during the interviews, participants were asked to complete NASH-CHECK while “thinking aloud” during the process, to help the interviewer to understand the thought processes behind participants’ responses and also to identify any apparent problems or hesitations in completing the measure [23, 24]. The interviewer then asked questions designed to elicit participants’ overall thoughts on the questionnaire and their views on the meaning and clarity of questionnaire content, the length and layout of the questionnaire, relevance of the items to participants’ experiences, and any missing areas. At the close of the interviews, participants were asked if they had any further comments.

### Analyses

Thematic analysis was used to analyse the transcript data from the CE interviews. Analysis was conducted in parallel for US and UK data, with the research teams working in constant communication with each other. LD and JT acted as coders for the US data and MB and LMS for the UK data. Double coding was conducted by secondary coders on 10% of interview transcripts to ensure quality control. An initial coding frame, composed of a list of symptoms and broader HRQOL impacts, was developed from the interview guide. The coding frame was applied to the data by assigning codes to segments of text in the interview transcripts. Regular harmonization meetings were conducted between the coding teams at RTI-HS and Newcastle University to discuss emerging themes from the data. Although no major codes were added as a result of this process, subcodes were updated and applied repeatedly to both old and new transcripts as the analysis progressed. This process of reflection, review, and harmonization support the trustworthiness of the qualitative research. Analyses were conducted to ensure that saturation of coding content (limited new codes during the final sets of interviews) was achieved across the UK and US data. The analysis process was facilitated by using qualitative analysis coding software (Atlas.ti version 7.5.16 [Scientific Software Development; Berlin, Germany] for the US interviews and NVivo version 14 [Lumivero, Denver, CO, USA] for the UK interviews).

Analysis of the CD interview data was conducted primarily using interview field notes, supplemented by the transcripts. The main goals of this analysis were to evaluate the comprehensiveness of the content of NASH-CHECK for patients with compensated cirrhotic MASH (F4); determine the relevance, acceptability, and clarity of NASH-CHECK content for UK and US participants; and determine whether the participant sample identified any consistent problems with NASH-CHECK. The analysis was conducted in parallel in the US and the UK. The US analysis was conducted by LD and JT, and the UK analysis by MB and LMS. The teams conducted regular harmonization meetings to review, reflect and agree the results of the analysis.

The participant characteristics from the CE and CD interviews were summarised using Microsoft Excel^™^.

## Results

### Concept elicitation interviews

#### Participant characteristics

The CE interview sample included 33 participants (US = 9, UK = 24), with a mean age of 64.3 years (Table 1). A majority of the sample was female (60.6%) and White (97.0%), and the mean BMI of the sample was 34.7. The participants reported numerous comorbid health conditions, most commonly hypercholesterolaemia (90.1%), type 2 diabetes (87.9%), obesity (87.9%), hypertension (69.7%), and hypertriglyceridemia (54.5%). More than half of participants reported the perceived severity of their MASH to be moderate, severe, or very severe (42.4%, 9.1%, and 6.1%, respectively).

#### Symptom concepts

##### Symptoms addressed by NASH-CHECK

**Fig. 2:**
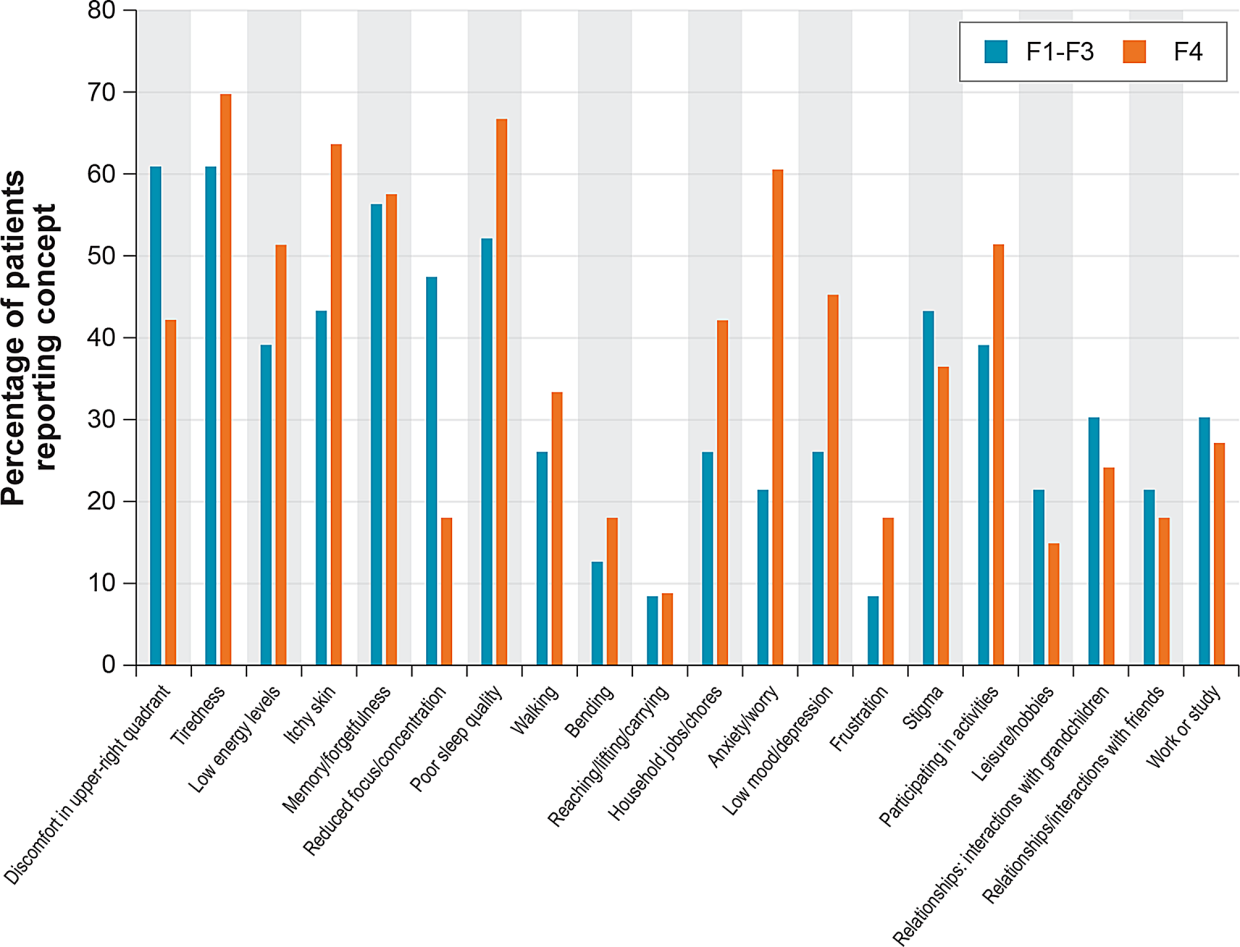
NASH-CHECK symptoms and HRQOL impacts reported by patients with F1-F3 and F4 MASH. Note: The figure presents the percentage of symptoms and broader HRQOL impacts included in NASH-CHECK that were reported by patients with compensated cirrhotic MASH (F4) in the current study (*n* = 33 participants in concept elicitation interviews) compared with patients with non-cirrhotic MASH (F1-F3) from the original NASH-CHECK development study (*n* = 23 participants in concept elicitation interviews). F = fibrosis stage; HRQOL = health-related quality of life; MASH = metabolic dysfunction–associated steatohepatitis

A summary of the coding saturation results is provided in Supplemental Appendix C (Table C1). The analysis indicated that saturation of key symptoms was reached during the analysis across the two samples. Three symptoms were identified in the final saturation group for the UK data, and six symptoms were identified in final saturation group for the US data. In the US sample, four of six symptoms were idiosyncratic issues raised by a single individual. All but one of the nice symptoms (“taking naps during the day” in the US data) identified in the final saturation groups for the US and UK samples were symptoms that were subsequently deemed not to be related to MASH based on expert review. All symptoms included in NASH-CHECK were reported by participants in the CE interviews. The symptoms reported most frequently by participants during the interviews were tiredness (69.7%), poor sleep quality (66.7%), and itching skin (63.6%) (Table 2). Participants also commonly reported experiencing symptoms such as abdominal pain and bloating in the abdomen, shortness of breath, and feelings of forgetfulness. The frequency of symptom reports for the sample of interviewed patients with compensated cirrhotic MASH (F4) compared with the sample of patients with non-cirrhotic MASH (F1-F3) from the original development of NASH-CHECK is shown in Fig. 2. With the exception of ‘discomfort in the upper right quadrant’ and ‘reduced focus/concentration’, symptoms were reported more frequently by patients with compensated cirrhotic MASH than by those with non-cirrhotic MASH.

##### Additional symptoms identified

Sixteen additional symptoms that were not addressed by NASH-CHECK were described by participants during the CE interviews (Table 3)—most frequently, muscle cramps (54.6%), generalised pain (42.4%), and shortness of breath (42.4%). These additional symptoms were compared with the symptoms identified during CE interviews with patients with non-cirrhotic MASH (F1-F3) [11]. Five of the symptoms were identified during previous development work with patients with non-cirrhotic MASH (F1-F3) and were reviewed by clinical experts in MASH but were deemed to be related to other health conditions rather than to MASH; therefore, they were not included in the final NASH-CHECK. The remaining 11 additional symptoms not previously identified during the CE interviews with the sample of patients with non-cirrhotic MASH (F1-F3) were reviewed in detail by a panel of clinical experts in the second phase of the study, as described below.

##### Clinical expert review of symptom impacts

Clinical experts reviewed the 11 additional symptoms identified during the CE interviews to determine whether amendments should be made to the content of NASH-CHECK to include these additional symptoms. The clinical experts determined that these symptoms (1) were not related to MASH, (2) were strongly associated with other comorbid conditions, or (3) were associated with decompensated cirrhotic MASH (F4) (Table 4).

#### Broader HRQOL impacts

##### Impacts addressed by NASH-CHECK

A summary of the coding saturation results is provided in Supplemental Appendix C. The coding analysis indicated that saturation of key HRQOL impacts was reached during the analysis; no HRQOL impacts were identified in the final saturation group for the UK data, and just one HRQOL impact was identified in the final saturation group for the US data. Of the activity limitation concepts addressed by NASH-CHECK, those most frequently reported by participants in the CE interviews were related to household chores (42.4%), walking (33.3%), and completing/pacing tasks (30.3%) (Table 4). Participants reported difficulty in walking, particularly over long distances and walking up stairs. Of the psychosocial concepts included in NASH-CHECK, those most frequently reported were anxiety/worry (60.6%), participating in activities (51.5%), and low mood/depression (45.5%) (Table 4).

Participants emphasised the emotional impact of MASH, particularly the effects of stigmatisation, feelings of embarrassment about personal appearance, and feelings of low mood. Participants reported that terminology associated with MASH can be misleading (e.g., the term *hepatitis*), which can result in patients with MASH being stereotyped as alcoholics. More than half of participants (60.6%) reported feeling anxiety because of MASH, expressing worry about the speed of disease progression and the potential need for a liver transplant in the future. Nearly one-fifth (18.2%) of participants reported a negative impact of MASH on relationships and interactions with friends, stating that their condition made them feel isolated and limited the activities in which they could participate. Changes in work patterns were reported by more than one-quarter (27.3%) of participants; only 18.2% of participants reported that they were still employed full time, with two additional participants working from home and another working part time. The rest of the participants were on long-term disability, retired, unemployed, or caring for family members at home.

The frequency of reports of overlapping, broader HRQOL impacts raised by the sample of patients with compensated cirrhotic MASH (F4) was compared with the frequency of mentions from the sample of patients with non-cirrhotic MASH (F1-F3) from the original development of NASH-CHECK (Fig. 2). In most cases, the broader HRQOL impacts were reported either more frequently by patients with compensated cirrhotic MASH or by a similar proportion of patients from each fibrosis group.

##### Additional impacts identified

Participants described 11 additional HRQOL concepts during the CE interviews that were not included in NASH-CHECK. Of these, seven concepts were identified previously in the CE interviews with patients with non-cirrhotic MASH (F1-F3) [11]. These concepts were not included in the original NASH-CHECK, either because they were removed during NASH-CHECK development for a F1-F3 patient population (e.g., concepts considered idiosyncratic or duplicate; concepts that presented variability in interpretation) or because they were removed during the psychometric analysis of NASH-CHECK (F1-F3 patient population) due to weak/unclear association with the other items. The remaining four additional HRQOL concepts were reviewed by the research team to determine whether any amendments to the content of NASH-CHECK were required. None of the additional concepts reported by participants with compensated cirrhotic MASH during CE interviews were added to the content of NASH-CHECK due to one or more of the following issues: the concepts were raised by four (12.1%) participants or fewer; there was a lack of relevancy for all participants (e.g., avoiding medications for comorbidities, concern about appearance); there was an anticipated lack of responsiveness to change (e.g., self-blame/regret); or the concepts overlapped with existing concepts in NASHCHECK (e.g., fear). Table 5 lists the 11 additional concepts that were evaluated and the reasons for their exclusion.

#### Conceptual model of MASH

**Fig. 3:**
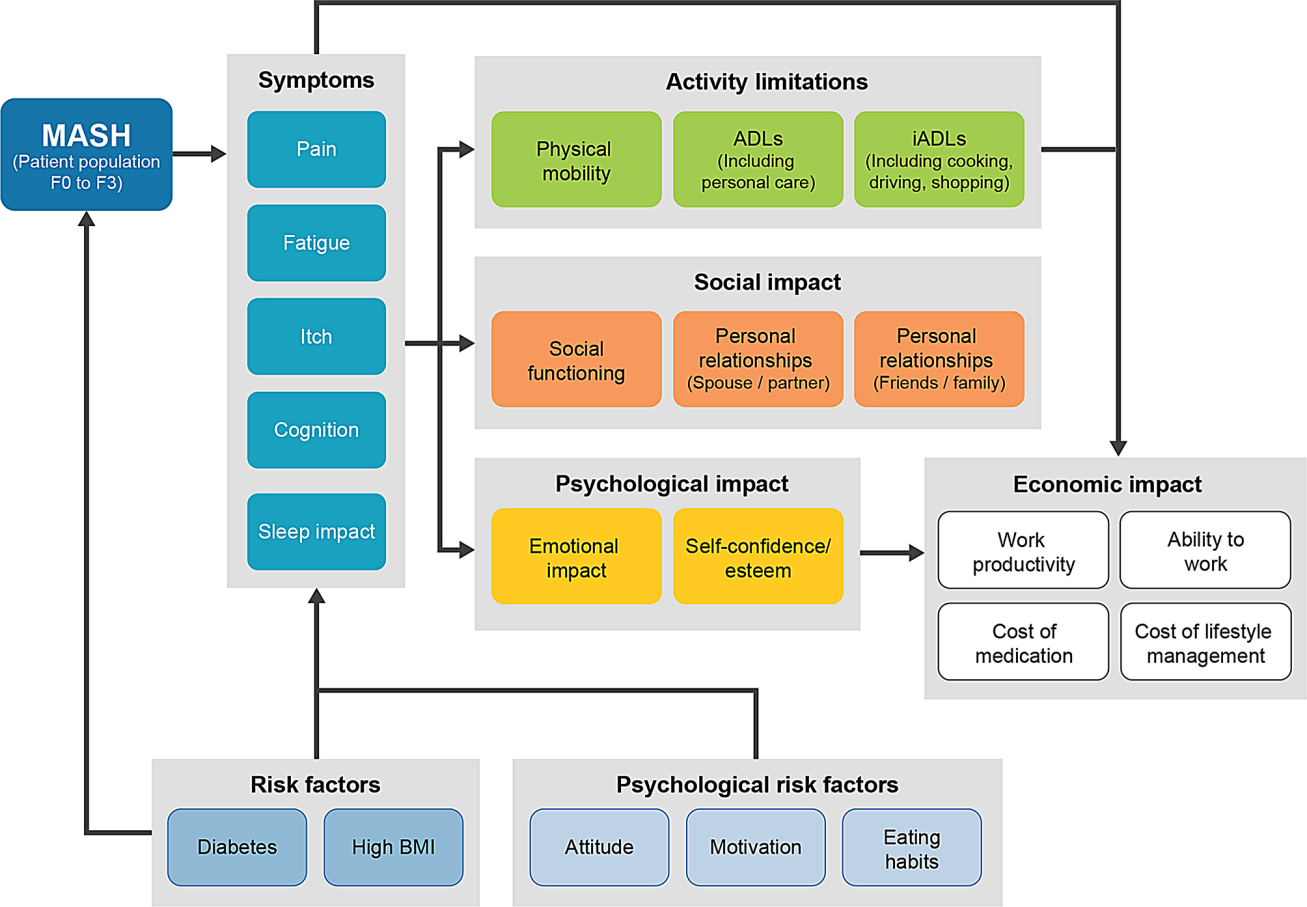
Conceptual model of MASH. ADL = activity of daily living; BMI = body mass index; iADL = instrumental ADL; MASH = metabolic dysfunction–associated steatohepatitis

Results of the CE interviews confirmed that the conceptual model developed for non-cirrhotic MASH is appropriate for patients with compensated cirrhotic MASH (Fig. 3).

### Cognitive debriefing interviews

The CD interview sample included 17 participants (US = 8, UK = 9), with a mean age of 62.8 years. Just under half of the sample was female (47.1%), and the mean BMI of the sample was 34.7. Participants reported the severity of their MASH to be mild, moderate, or severe (47.1%, 23.5%, and 29.4%, respectively). Information available on comorbid health conditions experienced by patients showed that obesity (88.2%), hypertension (88.2%), type 2 diabetes (82.4%), and hypercholesterolaemia (76.5%) were the most frequently reported.

Patient feedback on NASH-CHECK content confirmed that the symptom and broader HRQOL concepts captured were considered important, relevant, and comprehensive for addressing the impact of compensated cirrhotic MASH (F4) (Table 6). In addition, participants found the NASH-CHECK instructions, recall period, and response options clear and appropriate.

## Discussion

Understanding of the patient perspective on the impact of compensated cirrhotic MASH (F4) is limited by the use of PROMs that are inconsistent with current industry or regulatory standards or were not validated for a population with cirrhotic MASH [16]. For evidence presented to support novel therapeutic agents for licensing, regulatory authorities in the US and Europe require an evaluation of the therapy’s impact on how a patient ‘feels and functions’. The move towards patient-focused drug development has highlighted the need for well-targeted, validated PROMs to capture the patient perspective on (1) the experience of symptoms and (2) the wider impact of a disease on the patient’s HRQOL [25].

NASH-CHECK is a novel, disease-specific PROM developed according to industry and regulatory standards [20–22] to assess symptoms and broader HRQOL impacts of MASH in patients with non-cirrhotic MASH (F1-F3). The intention of the present study was to evaluate the suitability of this PROM for a compensated cirrhotic MASH (F4) patient population by assessing the symptom burden and other HRQOL impacts experienced by patients with compensated cirrhotic MASH and comparing these impacts with the impacts captured by NASH-CHECK.

All symptoms and HRQOL impacts included in the final version of NASH-CHECK were described by participants in the CE interviews, confirming that the conceptual model developed for non-cirrhotic MASH is appropriate for patients with compensated cirrhotic MASH. Of the additional symptoms and HRQOL impacts reported by participants with compensated cirrhotic MASH, none were deemed to be appropriate for inclusion in NASH-CHECK based on the assessments of clinical experts and the research team. Thus, no additions to NASH-CHECK were required, and the content of NASH-CHECK was considered appropriate for patients with compensated cirrhotic MASH. The final 28-item version of NASH-CHECK was evaluated further via CD interviews with participants with compensated cirrhotic MASH, and the results supported the content validity of NASH-CHECK for a cirrhotic MASH patient population.

Our findings indicate that patients with compensated cirrhotic MASH (F4) experience symptoms and HRQOL impacts similar to those experienced by patients with pre-cirrhotic MASH (F1-F3), with most issues being experienced with higher frequency and intensity in the compensated cirrhotic MASH population. In particular, compared with patients with non-cirrhotic MASH (F1-F3), those with compensated cirrhotic MASH reported greater frequencies of tiredness, low energy levels, poor sleep quality, and pruritus. This body of research confirms the continued relevance of a common set of core disease symptoms and broader HRQOL impacts throughout the disease continuum from non-cirrhotic to compensated cirrhotic MASH.

Some limitations of this study must be noted. A purposive sampling approach was used to identify the study participants; therefore, the sociodemographic characteristics of the CE and CD interview participants may not be reflective of the wider population of patients with compensated cirrhotic MASH. Nonetheless, the comorbidities participants reported reflect those generally seen in MASH populations worldwide [26]. Interview participants were eligible if they had histologically confirmed MASH cirrhosis or clinical suspicion of progression to MASH cirrhosis, so not all participants had biopsy-confirmed MASH. Participation in the interviews was entirely voluntary, and it is possible that such self-selection could also lead to selection bias. Because the interviews were semi-structured, the frequency with which each concept was reported reflects the number of times it was discussed as part of the interview, rather than the exact number of participants experiencing each issue. The reported frequencies may not precisely reflect the number of individuals experiencing each issue.

## Conclusion

Findings from this qualitative study suggest that the experiences and concerns of patients with compensated cirrhotic MASH are similar to the experiences and concerns of patients with pre-cirrhotic MASH (fibrosis stages F1 to F3). Results of the CE and CD interviews provide evidence to support the content validity and face validity of NASH-CHECK for compensated cirrhosis MASH patients, demonstrating that NASH-CHECK is a suitable PROM for use in clinical trials, non-interventional studies, and clinical practice for this patient population.

**Table 1:** Interview participant characteristics

Characteristic	CE sample (*n* = 33)	CD sample (*n* = 17)
**Age (years)**		
Mean (SD)	64.4 (7.8)	62.8 (7.9)
Median (Q1, Q3)	66.0 (60.0, 71.0)	63.0 (59.0, 70.0)
Min, max	47.0, 75.0	43.0, 72.0
Missing, n (%)	1 (3.0)	0
**Sex, n (%)**		
Female	20 (60.6)	8 (47.1)
Male	13 (39.4)	9 (52.9)
**Relationship status, n (%)**		
Married/living as married/civil partnership	24 (72.7)	14 (82.4)
Widowed/surviving partner	6 (18.2)	0 (0.0)
Divorced/separated	2 (6.1)	2 (11.8)
Single	1 (3.0)	1 (5.9)
**Ethnicity, n (%)**		
White	32 (97.0)	17 (100.0)
Black/African American	1 (3.0)	0 (0.0)
Other^ a^	0 (0.0)	0 (0.0)
**Employment status, n (%)**		
Working full time	6 (18.2)	3 (17.6)
Working part time	1 (3.0)	1 (5.9)
Retired/semi-retired	20 (61.0)	8 (47.1)
Long-term sick leave or disability	3 (9.0)	1 (5.9)
Unemployed	1 (3.0)	4 (23.5)
Work at home providing care for child/family	2 (22.2)	0 (0.0)
**Years since MASH diagnosis**		
Mean (SD)	5.5 (3.9)	6.7 (7.8)
Median (Q1, Q3)	4.8 (3.3, 7.5)	3.3 (1.3, 8.0)
Min, max	0.2, 17.0	0.7, 27.9
**Years since cirrhosis diagnosis**		
Mean (SD)	3.8 (2.5)	3.2 (2.6)
Median (Q1, Q3)	3.7 (2.3, 5.1)	3.2 (1.1, 4.1)
Min, max	0.2, 9.4	0.7, 10.4
Missing, n (%)	0 (0.0)	2 (11.8)
**Other health conditions, n (%)** ^**b**^		
Type 2 diabetes	29 (87.9)	14 (82.4)
Obesity	29 (87.9)	15 (88.2)
Hypercholesterolaemia	30 (91.0)	13 (76.5)
Hypertension	23 (70.0)	15 (88.2)
High triglycerides	18 (54.6)	6 (35.3)
Depression	10 (30.3)	5 (29.4)
Coronary heart disease	7 (21.2)	3 (17.6)
Cholecystectomy	5 (15.2)	1 (5.9)
Metabolic syndrome	5 (15.2)	1 (5.9)
**BMI**		
Mean (SD)	34.7 (4.5)	34.7 (4.4)
Median (Q1, Q3)	34.8 (31.6, 37.5)	34.5 (31.9, 37.1)
Min, max	25.6, 45.0	26.2, 43.1
**Obesity class, n (%)**		
1	13 (39.4)	8 (47.1)
2	13 (39.4)	5 (29.4)
3	3 (9.1)	2 (11.8)
**MELD score**		
Mean (SD)	7.3 (1.2)	6.9 (1.1)
Median (Q1, Q3)	7.0 (6.7, 8.3)	7.0 (6.0, 7.0)
Min, max	6.0, 9.0	6.0, 9.0
Missing, n (%)	21 (63.6)	10 (58.8)
**Child-Pugh score**		
Mean (SD)	5.3 (0.5)	5.2 (0.4)
Median (Q1, Q3)	5.0 (5.0, 6.0)	5.0 (5.0, 5.0)
Min, max	5.0, 6.0	5.0, 6.0
Missing, n (%)	21 (63.6)	11 (64.7)
**General health, n (%)**		
Very good	3 (9.1)	1 (5.9)
Good	12 (36.4)	8 (47.1)
Fair	16 (48.5)	3 (17.6)
Poor	2 (6.1)	5 (29.4)
**MASH severity, n (%)**		
Mild	12 (36.4)	8 (47.1)
Moderate	14 (42.4)	4 (25.5)
Severe	3 (9.1)	5 (29.4)
Very severe	2 (6.1)	0 (0.0)
Missing	2 (6.1)	0 (0.0)
**Life impact due to MASH, n (%)**		
A lot	5 (15.2)	4 (23.5)
Quite a lot	5 (15.2)	2 (11.8)
A little	12 (36.4)	6 (35.3)
Not at all	10 (30.3)	4 (23.5)
Missing	1 (3.0)	1 (5.9)

BMI = body mass index; CD = cognitive debriefing; CE = concept elicitation; MASH = metabolic dysfunction–associated steatohepatitis; MELD = Model for End-stage Liver Disease; Q = quartile; SD = standard deviation

^a^ Detailed additional categories provided in demographic questionnaire have been omitted from the table as no responses were given for these

^b^ Only conditions reported by five or more patients are reported

**Table 2:** Concept elicitation interviews: frequency of reported symptoms included in NASH-CHECK

Symptom	Frequency reported by sample with compensated cirrhotic MASH, *n* (%)^ a^	Keywords/phrases	Illustrative participant quote
Tiredness	23 (69.7)	♣ Tired ♣ Fatigue ♣ Tiredness ♣ Sleepy	“I’m worn out.” “The last few days, I have felt tired and have been in bed by half 7:00.”
Poor sleep quality	22 (66.7)	♣ Wakes you up ♣ Difficult to get to sleep ♣ Don’t sleep well ♣ Waking up	“I mean, because when it hurts, every move I make it just kind of catches. And it, you know, wakes you up if you go to sleep.” “Last night and the night before, I only slept part of the time.” “I feel a bit tired through the day because I don’t sleep a lot at night.”
Itching skin	21 (63.6)	♣ Itching ♣ Night-time ♣ Itchy ♣ Scratch	“It used to be extremely itchy.” “My back itches all the time, and my stomach.” “I’m scratching all the time. I’ve had to buy back scratchers and all sorts. And you’re scratching the same spot for 5 min, and it just won’t go away.”
Memory/forgetfulness	19 (57.6)	♣ Memory ♣ Retention ♣ Forget ♣ Remember ♣ Foggy	“Yeah, foggy. Just, you know, like I forget—not things, but I forget where I am in a sentence.” “In daily stuff, there’s some words that will disappear.” “I used to be able to remember everything. Now I’m finding I can’t remember half the things. I’m not as quick to pick things up. I’m thinking, ‘What is going on here?’”
Low energy levels	17 (51.5)	♣ Exhausted ♣ Energy ♣ Low energy ♣ No energy	“I can’t do the things that I used to do.” “I’m finding out…like cleaning the house, I cannot clean the whole house anymore.” “It’s as if I’ve got fatigue, no energy. I can’t really do very much at all.”
Abdominal pain	14 (42.4)	♣ Chronic upper-right region pain ♣ Stomach ♣ Abdominal pain ♣ Right-upper-quadrant discomfort ♣ Pressure ♣ Discomfort ♣ Inflammation	“I have always had this pain in my upper-right side.” “Sometimes, though, my liver is inflamed, you know, and it hurts.” “Well, this week I had a lot of pain on this side—niggling like, but quite sore.”
Abdominal swelling	7 (21.2)	♣ Bloating ♣ Discomfort	“…feeling of just being bloated and kind of this uncomfortable…” “I have bloating, but then I put that down to eating too much bread.”
Increased sleep	6 (18.2)	♣ Low mood ♣ Sleepy	“I’ve gotten to the point I could sleep all day and all night.” “Not knowing…is tomorrow going to be a good day or am I going to sleep all day.” “Now I can sleep the clock around if I want, so I do sleep a lot.”
Reduced focus	6 (18.2)	♣ Focusing ♣ Concentration ♣ Clear ♣ Slow ♣ Foggy	“There was no mental clarity there.” “I can’t concentrate. I can’t think straight. There’s like a fog.” “It’s hard to explain, that one. But sharpness, probably I’m not as sharp as I used to be when I was younger.”
Dry skin	2 (6.1)	♣ Dryness ♣ Itchy	“That whole thing right there itches like crazy.”

^a^ Frequency denotes the number (%) of participants who reported the issue

**Table 3:** Concept elicitation interviews: new symptom concepts identified and reasons for omission from NASH-CHECK

Compensated cirrhotic concept	Frequency reported by sample with compensated cirrhotic MASH, *n* (%)^ a^	Identified in the non-cirrhotic MASH (F1-F3) CE analysis	Reason for omission from NASH-CHECK during development for the non-cirrhotic MASH (F1- F3) population	Clinical evaluation
Pain: muscle cramps	18 (54.6)	NR	NA	♣ No agreement reached on the relevance of muscle cramps to MASH cirrhosis ♣ Noted that muscle cramps may be related to age
Pain: other non-abdominal	14 (42.4)	NR	NA	♣ Considered a comorbidity ♣ Noted that this may be related to age or other comorbid factors
GI: heartburn/GERD	13 (39.4)	Concept identified	Considered a comorbidity following clinical evaluation	NA
GI: constipation	6 (18.2)	Concept identified	Considered a comorbidity following clinical evaluation	NA
GI: nausea/vomiting	5 (15.2)	Concept identified	Considered a comorbidity following clinical evaluation	NA
GI: diarrhoea	4 (12.1)	Concept identified	Considered a comorbidity following clinical evaluation	NA
GI: gastroparesis	2 (6.1)	NR	NA	♣ Considered a comorbidity ♣ Usually a complication of diabetes ♣ It was noted that gastroparesis is a clinical sign rather than a patient-perceived symptom, so would not be suitable for inclusion in a PRO
GI: gastric antral vascular ectasia	1 (3.0)	NR	NA	♣ Considered a comorbidity ♣ GI issues are common in the general population
Cardiovascular: shortness of breath	14 (42.4)	Concept identified	Considered a comorbidity following clinical evaluation	NA
Cardiovascular: anaemia	3 (9.1)	NR	NA	♣ Considered a comorbidity ♣ It was noted that anaemia is a clinical sign rather than a patient-perceived symptom, so anaemia would not be suitable for inclusion in a PRO
Cardiovascular: oedema/swollen extremities	2 (6.1)	NR	NA	Considered a comorbidity
Cardiovascular: numbness in extremities	1 (3.0)	NR	NA	Considered a comorbidity
Increased infections	4 (12.1)	NR	NA	♣ Considered a comorbidity ♣ This was not considered to have relevance in an outcome measure
Dizziness	5 (15.2)	NR	NA	♣ Could be a sign of decompensation or caused by diuretic medication
Variceal bleeding (vomiting blood)^ b^	3 (9.1)	NR	NA	♣ Symptom of decompensation
Increased allergies	1 (3.0)	NR	NA	♣ Considered a comorbidity

CE = concept elicitation; F = fibrosis stage; GERD = gastroesophageal reflux disease; GI = gastrointestinal; MASH = metabolic dysfunction–associated steatohepatitis; NA = not applicable; NR = not reported; PRO = patient-reported outcome

^a^ Frequency denotes the number (%) of participants who reported the issue

^b^ Identified as a past symptom

**Table 4:** Concept elicitation interviews: frequency of reported HRQOL impacts included in NASH-CHECK

Concept	Frequency, *n* (%)^ a^	Keywords/phrases	Illustrative participant quotes
**Day-to-day activities**			
Household jobs/chores	14 (42.4)	♣ Vacuum ♣ Sweep the floor ♣ Change lightbulbs ♣ Gardening	“I’m finding out…like cleaning the house, I cannot clean the whole house anymore.” “I can’t do the things that I used to do.” “I used to do a lot of cooking. I can’t do much of that now. I might do a little bit of salad for myself, but I’m absolutely shattered.”
Walking	11 (33.3)	♣ Walk ♣ Walk 5 miles a day ♣ Up and down stairs	“I used to walk with my friends in the evenings, but now it hurts to walk.” “I had to find a ranch house to move to because I can’t do stairs.” “I do have a lot of trouble walking around. I keep saying I am going to apply for a blue badge because even getting around the house is difficult.”
Completing/pacing tasks	10 (30.3)	♣ Take breaks ♣ Rest	“I try to do a little bit and then rest, do a little bit, rest; and then it’s like by the time I get to doing something else, that first thing gets all messed up again.” “I just try and limit, you know, do a little bit at a time.”
Bending over	6 (18.2)	Bending over to: ♣ Tie shoes ♣ Put shoes or socks on	“It hurts my stomach.” “I can’t bend well. It’s very painful.”
Lifting	6 (18.2)	♣ Carrying ♣ Lifting	“I feel like I can’t keep up with stuff, you know. Lifting, you know.” “I can’t lift heavy things anymore. I do try, but I find I can’t, so I give up as a bad job.”
Physical activities	4 (12.1)	♣ Less activity ♣ Less going outside ♣ Pain	“Just when I have those bad days of pain. Then I just really just don’t feel like doing anything.” “Now when I’m having the discomfort, I don’t feel like doing anything much.”
Shopping	1 (3.0)	♣ Less going shopping	“I take it easy, you know, until [the pain] abates.” “I will do some things because I have to go out. I have to shop, but oh, sometimes, I’m so exhausted of that, I don’t even take the shopping out of the car.”
**Psychosocial concepts**			
Anxiety/worry	20 (60.6)	♣ Worry ♣ Anxious ♣ Anxiety	“I guess I keep worrying about it, and I keep asking [name deleted]: do I need to be on a liver transplant list or something?” “I feel as if I don’t know what is happening, so I am concerned about the fact that I don’t know what is happening. And maybe I am getting too obsessed with it and looking at things and looking up things to see if this is happening and that is happening. So it’s taking over, really, a bit.”
Participating in activities	17 (51.5)	♣ Social drinking ♣ Can’t get out today ♣ Don’t really feel like socialising ♣ Limits activities	“It pretty much messes my day up because I’m pretty much home. I try not to go and do anything on those days and always reschedule.” “I think I’ve become more withdrawn, as well, so socialising. I don’t socialise as much. I don’t go out as much. So I used to go out quite a lot. Now I just tend to stay in the house and go out now and then.”
Low mood/depression	15 (45.5)	♣ Depressing ♣ Moodiness ♣ Depression ♣ Depressed	“Depression, you know, moodiness, just feeling like crap.” “Well, on days I don’t feel good, I’m kind of down.” “When I was first diagnosed, I had a few tears when I got home, and I’ve never cried about it since. But there used to be big trees just behind those houses. They’re building there now, but I used to look at those, and I used to sometimes say when I sat over there, ‘I wonder if I’ll be here when they fall, or will I be here next summer when they come out in spring?’ And when I got my great-grandchildren, I used to sometimes think, ‘I’m not going to be here indefinitely, not going to be around to see them growing up.’ So I make the best of it now.”
Stigma	12 (36.4)	♣ Feel judged ♣ It is not hepatitis ♣ Stereotype me as a drinker ♣ They don’t physically see something ♣ I didn’t want people to think I was an alcoholic	“When you tell them you’ve got cirrhosis, they automatically think you’re an alcoholic.” “You know how people don’t, they see you, and they think, ‘Oh, you’re fat, you know; I see you do this, this.’ Yeah, if you’re on disability, you shouldn’t be, you know…They don’t know how I feel inside.” “As soon as you mention the word cirrhosis, people think drink. ‘His liver must be pickled.’ That’s the way most people think. I think it’s through not knowing that there is another cirrhosis if you like.”
Changes in work pattern/ ability	9 (27.3)	♣ Early retirement ♣ Lack of energy ♣ Struggling to control symptoms	“I had my own business, and I just recently…I gave that up. I sold it, and now I don’t work.” The time off [from directing local charitable organisation] has to happen anyway because I have to allow space for the new director to come in. But I have actually extended that time off a bit because I would have to take 3 months.”
Relationships/interactions with friends	6 (18.2)	♣ Isolated ♣ Misunderstood ♣ Left out	“You know, I’m a people person, but I have found that I’ve gotten to where now I think I hope I don’t run into anybody I know. That’s bad to say, but I mean, it’s like I just don’t feel like socialising.” “They feel uncomfortable being around me. I don’t feel uncomfortable being around them.”
Leisure/hobbies	5 (15.2)	♣ Shopping ♣ Walking ♣ Socialising	“I used to be one that could go out and shop all day long. I’ll shop for a couple of hours, and I’m ready to hang it up.” “I used to like to do a lot of craft things, but I just don’t have the interest at the moment.”
Concentration on tasks	2 (6.1)	♣ Lack of mental clarity	“I just couldn’t do it. There was no mental clarity there.”

HRQOL = health-related quality of life

^a^ Frequency denotes the number (%) of participants who reported the issue

**Table 5:** Concept elicitation interviews: new HRQOL concepts identified and reasons for omission from NASH-CHECK

Compensated cirrhotic concept	Frequency reported by sample with compensated cirrhotic MASH(%)^ a^	Identified in the non-cirrhotic MASH (F1-F3) CE analysis	Reason for omission from NASH-CHECK during development for the non-cirrhotic MASH (F1-F3) population	Additional evaluation
Perceived burden	4 (12.1)	Concept identified Item included in draft 1 of NASH-CHECK: “I feel like I am a worry to my family”	Removed following psychometric evaluation due to weak/unclear associations with other items	NA
Restrictive/bland diet	4 (12.1)	Concept identified Item included in draft 1 of NASH-CHECK: “I feel restricted in the foods I can eat”	Removed following psychometric evaluation due to weak associations with other items	NA
Psychosocial: fear	11 (33.3)	NR	NA	Considerable overlap with the “worry” item (Item 18) already included in NASH-CHECK
Psychosocial: self-blame/regret	7 (21.2)	NR	NA	Reported by relatively few participants Considered not to be a suitable measure of health outcome, as it is unlikely to be responsive to change in condition
Psychosocial: irritability	4 (12.1)	Concept identified Item included in draft 1 of NASH-CHECK: “I feel irritable because of my fatty liver disease”	Removed after CDIs Considered to be an irrelevant concept	NA
Psychosocial: difficulty coping with major life events	3 (9.1)	NR	NA	Reported by relatively few participants Considered not to be a suitable health outcome measure, as the concept may not be relevant or applicable to all participants
Psychosocial: appearance (concerns about body shape/ability to make clothes choices)	1 (3.0)	Concept identified 2 items included in draft 1 of NASH-CHECK: “I am concerned about the way my illness affects my appearance” and “My abdominal (stomach) bloating affects what clothes I can wear”	Removed after CDIs Not relevant to all, and variation in understanding observed	NA
Diet: healthy eating choice	23 (69.7)	Concept identified Item “I find it difficult to plan healthy meals” included in draft 1 of NASH-CHECK	Removed after CDIs Not relevant to all participants, and variability in interpretation as either a positive or a negative issue	NA
Diet: avoiding alcohol	15 (45.5)	Concept identified No item constructed	Not applicable to all participants	NA
Diet: avoiding medications for comorbidities	5 (15.2)	NR	NA	Reported by relatively few participants Not applicable to all participants
Relationship: hiding disease symptoms and impact from others	9 (27.3)	Related concepts identified 2 items in draft 1 NASH-CHECK: “I try to hide my fatty liver disease from others” and “I worry about telling people about my fatty liver disease”	Removed after CDIs Duplication of concepts, and not clearly understood by all participants	NA

CDI = cognitive debriefing interview; CE = concept elicitation; F = fibrosis stage; HRQOL = health-related quality of life; MASH = metabolic dysfunction–associated steatohepatitis; NA = not applicable; NR = not reported

**Table 6:** Key findings from the cognitive debriefing interviews (Combined UK and US sample *n* = 17)

Section	Summary of findings	Illustrative participant quotes to support understandability and relevance
Overall impression of questionnaire	♣ Reported to be clear, comprehensive and relevant to the participant experience of MASH	♣ “Very easy to follow which was nice… and then it covered everything”
Questionnaire title and instructions	♣ Reported to be clear and suitable by most participants ♣ Instructions were understood clearly	♣ “Yes they [the instructions] were quite clear.” ♣ “I thought it [the title] was fine and it was clear and I know what I’m into with this.”
**Symptoms**		
Instructions	♣ Instructions were understood clearly by most participants.	♣ “Yes, they [the instructions] were fine; they were easy to understand.” ♣ “[The instructions] very easy to follow which was nice.”
Recall period: past 7 days	♣ Most participants found the recall period appropriate and easy to use when answering the questions.	♣ “You know, in the past 7 days let me know exactly what you’ve been experiencing” ♣ “Yeah, I think it [past 7-days recall] works very well” ♣ [How easy or hard it is to think back 7 days ago to answer the questions]… “that’s not hard at all.”
Response options: 11-point NRS	♣ Generally, participants found the response scale clear and easy to use.	♣ **“**Yes, I had no problems keeping up that, yes sir, I could read that and understand it.” ♣ **“**[You felt like that was an appropriate number of response choices there? ]…yes”
	♣ Item considered clear	♣ “I have a pain on the upper right side where my liver is and I do, so it was very easy for me to answer that question.” ♣ [Helpful to include the term stomach area to identify the pain location?”]…“Yes.”
Item 2: Abdominal bloating	♣ Item considered clear	♣ “Well, it says bloating in your stomach would feel like it was overfull or in discomfort or couldn’t get comfortable in general” ♣ “Yes, it’s very much relevant of…there’s a lot of the bloating.” ♣ “I think that’s [the word bloating] fairly clear and I know that I’ve felt that at times, so that, to me, absolutely fine.”
Item 3: Fatigue	♣ Item considered clear	♣ “I have noticed a lot of fatigue.” ♣ “Fatigue is tiredness, do I feel you can’t be bothered.” ♣ [Fatigue means] “I can’t put one foot in front of the other without going…you know, just holding onto the side of the wall and just feeling like I just can’t…I can’t participate in life this day.”
Item 4: Sleep	♣ Item considered clear ♣ Several participants reported sleep problems	♣ “I could understand [the question] that without any issues…” ♣ “That’s quite clear to me and I have no difficulty sleeping at all.” ♣ “The difficult part is knowing that I’m going to go to bed and I’m going to be up at 3:00 o’clock awake… I can’t go back to sleep.”
Item 5: Focusing	♣ Item considered clear	♣ “sometimes I, um, tend to either forget a little bit or not remember all the details and certain things and so that the focus is not bad but it’s not 100%”. ♣ “it [item 5] was easy to understand, I knew what you were talking about” ♣ “Basically, it’s [item 5] fairly clear as far as I’m concerned.”
Item 6: Thinking clearly	♣ Item considered clear♣	♣ “I understand it [item 6] too, it’s good, it’s a good question.” ♣ [Difficulty thinking clearly means]… “Jumbled thoughts. And when you’re trying to plan a course of action which I need to do on a fairly regular basis.” ♣ [Difficulty thinking clearly means]… “Well saying part irrational things and going off on a tangent” ♣ [Difficulty thinking clearly means]… “Like you’re in a fog.”
Item 7: Following conversation	♣ Item considered clear♣	♣ “Conversations in general, do I have any problems keeping up or understanding and no, I don’t.” ♣ “Again, it’s [item 7] to me, fairly clear and straightforward.”
Item 8: Forgetful	♣ Item considered clear	♣ [Item 8 means]… “are you forgetful and sometimes I do forget little things [inaudible] like I’ve said before…” ♣ “Forgetful is forgetting things.” ♣ “I write most of my stuff down, and I started doing that a couple of years back, but I still do forget things” ♣ “Just not forgetful to do the important things… but forgetful like I said about, um, where I put something.”
Item 9: Itchy skin	♣ Item considered clear	♣ “I absolutely know what you’re talking about as far as the itchy skin and the skin problems…” ♣ “Yeah, my back is always itchy”
**Day-to-Day Activities**		
Instructions	♣ Instructions were understood clearly	♣ “Yeah, they were very easy to understand, and I could understand exactly what they were asking” ♣ [Wording and position of instructions] “Again, I thought all the way through it was fairly clear.”
Recall period: past 7 days	♣ Recall period was reported as being suitable by most participants	♣ [thinking about how easy or hard it is to think back 7 days ago to answer the questions] “Very easy. I had no problem with them whatsoever.”
Response options: 5-point Likert-type scale	♣ Reported to be easy to use and suitable	♣ “I thought they covered everything. They gave you the option of exactly where do you fall in there at.” ♣ “Very easy [to use].”
Item 10: Bending	♣ Item considered clear ♣ Examples received positive feedback♣	♣ “Yes, they’re straightforward, putting your shoes on or picking anything up.” ♣ “Okay. Bending over, in other words to put on your socks and shoes or to pick something up from the ground.” ♣ “Um, that [bending] is always a moderate difficulty… because when I bend over, it pinches and hurts that side.” ♣ “bending, the only thing that bothers me is the little bit of pain in here sometimes”
Item 11: Light chores	♣ Item considered clear ♣ Examples received positive feedback	♣ [Are the examples helpful? ]… “Yes.” ♣ [Item] “11 is clear” ♣ “The dishes pile up once in a while, and I’ll look at it and say, ‘You know, I just don’t feel like doing that right now,‘”
Item 12: Heavy chores	♣ Item considered clear ♣ Examples received positive feedback	♣ [Clarity / understandability] “Very much so, very much” ♣ “Doing heavy chores around the house in other words changing bed linens, vacuuming, taking the trash out and heavy gardening” ♣ “Changing bed linens, vacuuming, taking trash out, heavy gardening. Um, that’s moderate difficulty [for me].” ♣ [Helpfulness of examples]… “Yes, they’re okay, definitely.”
Item 13: Lifting	♣ Item considered clear ♣ Examples received positive feedback	♣ “Easy to understand and relate [to]” ♣ “I do not pick anything up other than something like a gallon of milk. So I have moderate difficulty as far as, um, lifting things”.
Item 14: Short walk	♣ Item considered clear ♣ Example received positive feedback	♣ [Helpfulness of example]… “Yes, yes, you know what a [short] walk is.” ♣ [Helpfulness of example]… Yes, very helpful, yes.
Item 15: Long walk	♣ Item considered clear ♣ Example received positive feedback	♣ [Helpfulness of example]… “Yes”
Item 16: Brisk walk	♣ Item considered clear	♣ [Brisk walk means] Um…one where I could make…it might be hard to breathe… ♣ [Brisk walk means] “…elevating my heart level up.” ♣ “It is clear what it actually means, yeah.”
Item 17: Walk up stairs	♣ Item considered clear	♣ “*I can climb like seven or eight at one time*,* but after that I need to stop and rest*…” ♣ [Is the meaning of ‘flight of stairs’ clear? ]… “yeah” ♣ [Interpretation of ‘flight of stairs’]… “ten or twelve”
**Emotions and Lifestyle**		
Instructions	♣ Instructions were understood clearly	♣ “Yeah, you’ve got five [options] here and again, it’s fairly clear.” ♣ “Yes, I can understand them well. Easy to use.”
Recall period: past 7 days	♣ Most thought recall period was suitable	♣ “It’s simple enough to understand over the last seven days.” ♣ [Is 7 days an appropriate recall? ]… “I do think so.”
Response options: 4-point Likert-type scale	♣ Easy to use and suitable	♣ “I think it covered everything, and I think it gave you the options to really tell how you are, and it affects you, and I think it did really well.” ♣ “I thought that was clear.”
Item 18: Worry	♣ Item considered clear	♣ “I did, yes.”
Item 19: Feel down	♣ Item considered clear	♣ “Yes, it was easy to understand, yes.”
Item 20: Feel angry	♣ Item considered clear	♣ “I understood it very well.” ♣: “I mean, the thing is the wording is, it’s very clear…”
Item 21: Feeling judged	♣ Item considered clear	♣ “…that is the right sort of question [item 21] to ask… but you do get judged by people. Nine times out ten, they won’t say it, but it’s in their head.” ♣ “I don’t really tell many people that I have it because I’m a little self-conscious.”
Item 22: Relationships	♣ Item considered clear	♣ “Yes, I did, easy to understand.” ♣ “Yeah, it does [affect my relationships”.
Item 23: Everyday activities	♣ Item considered clear	♣ “[Again, was that [item 23] easy to understand? ] Yes, very” ♣ “My seven friends, um, just went to [social event]…and I was the only one that didn’t get invited. And I know it was because…I would have held them back…from having their wildness. My feelings were hurt a little bit, but at the same time, they would have been putting me in the same, um…in a situation that I didn’t want to be in either.”
Item 24: Family life	♣ Item considered clear	♣ “Yes sir, very easy to understand that.” ♣ [Interpretation]… “just being able to,…go to see people.”
Item 25: Intimacy	♣ Item considered clear	♣ [Interpretation]… “About just being intimate and having a relationship or a normal relationship with my spouse.” ♣ “It [item 25] covers everything you need to cover, in my opinion”
Item 26: Socialise	♣ Item considered clear	♣ “I think that’s perfectly worded as it is as well.” ♣ “And that [item 26] referred me back to…being able to do things with friends…
Item 27: Spare time	♣ Item considered clear	♣ “I don’t do things like I used to do anymore, you know, as far as hobbies and things that I like to do.”
Item 28: Work or study	♣ Item considered clear	♣ It affects the ability to work and study…” ♣ “I think that [item 28] is good because that’s when you’re doing work and studying, you’ve got to use your mind, you’ve got to concentrate…”

NASH = non-alcoholic steatohepatitis; NRS = numeric rating scale

## Electronic supplementary material

Below is the link to the electronic supplementary material.

Supplementary Material 1

Supplementary Material 2

## Data Availability

Data are not available.

## Abbreviations

ADL: Activity of daily living
ALT: Alanine aminotransferase
AST: Aspartate aminotransferase
BMI: Body mass index
CD: Cognitive debriefing
CDI: Cognitive debriefing interview
CE: Concept elicitation
EMA: European Medicines Agency
F: Fibrosis
F: Fibrosis stage
FDA: Food and drug administration
GERD: Gastroesophageal reflux disease
GI: Gastrointestinal
HRQOL: Health-related quality of life
iADL: Instrumental activity of daily living
MASH: Metabolic dysfunction–associated steatohepatitis
MASLD: Metabolic dysfunction–associated steatotic liver disease
MELD: Model for End-stage Liver Disease
NAFLD: Non-alcoholic fatty liver disease
NASH: Non-alcoholic steatohepatitis
NA: Not applicable
NHS: National Health Service
NR: Not reported
NRS: Numeric rating scale
PRO: Patient-reported outcome
PROM: Patient-reported outcome measure
Q: Quartile
SD: Standard deviation
UK: United Kingdom
US: United States
