# Supplementary material for: NASH-CHECK patient-reported outcome instrument: evaluation of content and face validity for patients with metabolic dysfunction–associated steatohepatitis and compensated cirrhosis
Source: J Patient Rep Outcomes. 2025 Jul 1;9:76. doi: 10.1186/s41687-025-00881-6 (PMC12214096; doi:10.1186/s41687-025-00881-6)
Supplement: Supplementary file 1 — Supplementary Material 1 [file 41687_2025_881_MOESM1_ESM.docx]

# Supplemental Appendices

## Appendix A: Summary of Concept Elicitation Interview Guide

The interview guide included the following topics but remained flexible to allow patients to raise additional issues:

- Section 1: NASH history
- Impact of diagnosis
- Section 2: Patients’ experiences with NASH symptoms
- Types of symptoms
- Range of symptoms experienced
- Most important symptoms
- Section 3: Impact on day-to-day life
- Physical mobility
- Activities of daily living
- Instrumental activities of daily living
- Social life
- Work life
- Ability to sleep
- Healthy eating choices
- Section 4: Emotional impact
- Psychosocial impact
- Impact on mood
- Section 5: Impact on relationships
- Personal relationships
- Relationships with friends and family

## Appendix B: Summary of Cognitive Debriefing Interview Guide

Topics included in the cognitive debriefing guide were as follows:

- Section 1: Specific questions or follow-up from the think-aloud process related to:
- Reading opening and ongoing instructions
- Key comments or non-verbal cues
- Any items, or other aspects, associated with difficulty, uncertainty, confusion, hesitation
- Missing responses, answering in a different order, or answered with reference to another item
- Section 2: Review of NASH-CHECK
- Instructions
- Recall period
- Response options
- Individual items
- Section 3: General feedback on questionnaire
- Overall thoughts
- Length of the questionnaire
- Relevance to the participant’s experience
- Missing areas
- Layout of questionnaire

## Appendix C: Concept Elicitation Coding Saturation Results

Table C1. Concept Elicitation Coding Saturation Results for the UK and US Samples

| **Concept** | **UK unique codes** | | | | | **US unique codes** | |
| --- | --- | --- | --- | --- | --- | --- | --- |
|  | **Group 1 (n = 5)** | **Group 2 (n = 5)** | **Group 3 (n = 5)** | **Group 4 (n = 5)** | **Group 5 (n = 5)** | **Group 1 (n = 5)** | **Group 2 (n = 4)** |
| Symptoms | 17 | 4 | 8 | 0 | 3 | 24 | 6 |
| HRQOL | 14 | 7 | 7 | 3 | 0 | 28 | 1 |

HRQOL = health-related quality of life; UK = United Kingdom; US = United States.
